# Disparities in public transit access to healthcare in Austin, Texas

**DOI:** 10.3389/fpubh.2025.1689733

**Published:** 2025-11-03

**Authors:** Mei Yang, Tiankai Wang

**Affiliations:** ^1^Department of Geosciences, Mississippi State University, Starkville, MS, United States; ^2^Department of Health Informatics and Information Management, Texas State University, Round Rock, TX, United States

**Keywords:** public transit, healthcare access, geographic disparities, Black/African American, Hispanic/Latino, health insurance, poverty level

## Abstract

**Introduction:**

Unequal healthcare access is linked to disparities in health outcomes. Public transit plays a critical role in promoting equitable healthcare access, particularly for disadvantaged populations. This study aims to assess disparities in hospital access via public transit in Austin, Texas, while considering socioeconomic and demographic factors.

**Methods:**

We analyzed 30 hospitals using data from Definitive Healthcare, alongside demographic and socioeconomic factors for 283 census tracts in and around Austin, Texas, obtained from the U. S. Census Bureau. Variables included the percentage of the population who are Black or African American, Hispanic or Latino, uninsured, or have incomes below the poverty level. Using the TravelTime Isochrone API, we delineated one-hour public transit catchment areas for each hospital and overlaid them with demographic and socioeconomic data to examine spatial disparities in healthcare access and identify underserved communities.

**Results:**

Overall, people in the western and eastern parts of the city lack hospital service coverage accessible by public transit within 1 hour. Of the 283 census tracts, 160 are either partially covered (125 tracts) or not covered at all (35 tracts), with 72 of the partially covered tracts having less than 50 percent area coverage. The eastern area has higher proportions of Black or African American, Hispanic or Latino, and uninsured populations, reflecting greater disparities.

**Discussion:**

The results revealed notable disparities in healthcare access via public transit, where limited hospital coverage overlaps with high social and economic vulnerability. Targeted transit and healthcare planning for underserved areas and populations is needed to reduce these inequities.

## 1 Introduction

Promoting equality in healthcare access is essential, particularly for individuals in disadvantaged communities (1, 2). Unequal access to healthcare services is often associated with disparities in health outcomes, and the spatial patterns of illness are known to be related to the distribution of healthcare facilities (3, 4). Although healthcare facilities may be geographically available, reaching them remains a significant barrier for many individuals, which makes transportation an important factor in healthcare accessibility.

Public transit plays a crucial role in advancing equitable access to healthcare (5). It disproportionately serves low-income individuals, people with disabilities, minorities, and older adults, who often rely on public transit to reach essential services, including healthcare (6). In Greater Minnesota, Mattson et al. (7) reports that, on average, 17% of transit trips are for healthcare purposes. Additionally, 34% of respondents indicated they would have missed trips without transit services. Among the benefits of public transit, improved access to healthcare ranked highest in importance to users, emphasizing its essential role in supporting health equity.

Various studies have employed different methods to measure accessibility to healthcare facilities (8–15). Traditional approaches often relied on straight-line or Euclidean distances, which do not reflect actual travel conditions (8). More recent studies have adopted network-based models that account for real travel routes and times, offering a more accurate representation of accessibility (9). Building on these network-based concepts, several studies have leveraged Google Maps–based data and APIs to measure spatial accessibility at different geographic scales, providing near–real-time estimates of travel time and reflecting dynamic traffic and transit conditions relevant to healthcare access (10–12). Furthermore, studies employing cost-distance algorithms, such as the least-cost path algorithm implemented in AccessMod, have been widely used to model travel time and accessibility in diverse settings (12, 13). In the context of public transit, Hlusko et al. (14) constructed their own public transit network data and used a “Service Area” method to estimate the accessible area around a single hospital. Similarly, Ni et al. (15) utilized open APIs in China to estimate public transit travel times and integrated these with a door-to-door model to assess accessibility to community hospitals in Nanjing. Although these studies represent methodological advancements, their scope remains limited, as they either focus on a single hospital or do not account for broader social and economic disadvantages.

Existing literature has consistently highlighted that factors such as age (14), minority groups (4), income (16), and insurance coverage (17) significantly affect equitable access to healthcare. However, these dimensions are often overlooked in studies examining hospital accessibility via public transit. Additionally, there is a notable lack of research focused on public transit-based healthcare accessibility in Texas. For example, Maleki et al. (18) examined access to children’s hospitals but did not consider public transit access or broader population needs. This highlights a critical gap in understanding how public transit intersects with healthcare access in diverse and rapidly growing urban areas in Texas.

This study addresses these gaps by investigating public transit-based healthcare accessibility in Austin, Texas. Specifically, it aims to assess disparities in hospital access by integrating public transit network data with socioeconomic and demographic indicators. To achieve this, we examined the service coverage areas for all 30 hospitals, including healthcare centers, in Austin, and analyzed social and economic factors at the census tract level, including racial/ethnic composition (Black or African American, Hispanic or Latino), health insurance coverage, and income. Census tracts are small geographic units defined by the U. S. Census Bureau as subdivisions of a county, which generally have 1,200–8,000 people, with an optimum size of about 4,000 residents (19). The objective is to identify areas with vulnerable populations that face limited access to healthcare via public transit, thereby providing insights to inform more equitable urban and health planning.

## 2 Methods

### 2.1 Data source

Texas’s population is concentrated in major metropolitan areas, including Dallas–Fort Worth, Houston, Austin, and San Antonio, which are connected by a well-developed highway and toll road network (20, 21). Urban areas are primarily clustered along the Interstate 35 and Interstate 10 corridors, while rural regions dominate the western portion of the state. According to the U. S. Census Bureau’s urban–rural classification, approximately 83.7% of Texas residents live in urbanized areas (22). Within this broader context, we selected Austin, one of the most populous cities in Texas, as our study area due to its availability of complete public transportation (bus) stop data through the TravelTime API service (23). The city covers approximately 319.9 mi^2^ and has an estimated population of 961,855 residents (24). Of this population, 54.7% identify as White (non-Hispanic), 7.25% as Black or African American, 32.5% as Hispanic or Latino, 13% of the total population are without health care coverage, and the poverty rate remains at 11.8% (24). According to the Austin Strategic Mobility Plan (25), approximately 4% of commuters in Austin rely on public transit as their primary travel mode, indicating that a measurable segment of the population depends on public transportation for daily travel.

We included census tracts that are located within or intersect the Austin city boundary. For each tract, we collected demographic and socioeconomic data from the U. S. Census Bureau’s American Community Survey (ACS) 5-Year Estimates for 2023 (26). The data include the percentage of the population that is Black or African American, Hispanic or Latino, without health insurance coverage, and with income below the poverty level. Data on all types of hospitals in the study area was retrieved from the Definitive Healthcare database (27). These include short-term acute care hospitals, long-term acute care hospitals, psychiatric hospitals, rehabilitation hospitals, and children’s hospitals. In total, there are 30 hospitals and 284 census tracts in the study area, with one census tract lacking population data.

### 2.2 Catchment area analysis

As mentioned above, traditional catchment area analysis often relied on straight-line distance, which overlooked the realities of transportation networks and actual accessibility. In this study, we used a network-based approach to identify areas that were truly reachable within a specific time frame, rather than just those that were geographically close. We focused on hospital service areas accessible by public transportation, specifically bus transit, in Austin, Texas. Austin’s public transportation system is primarily bus-based and operated by Capital Metro, a regional public transportation agency that offers local routes, express busses, and ‘Rapid’ bus lines with transit-priority features along the city’s primary transportation corridors. Although the region also operates one commuter rail line, bus transit provides the primary coverage and ridership, and was therefore the focus of this study.

We used the Isochrone API from TravelTime (28), which enabled catchment area analysis based on travel time rather than distance. This API generates reachable areas within specified travel-time thresholds by simulating realistic routes along the underlying transport network. Its public transit modeling framework integrates multimodal data sources, including road networks, walking paths, and public transit schedules, combined with both historical and current traffic information (29). Rather than relying solely on real-time vehicle probe data, the system applies internally calibrated transport models that incorporate typical travel speeds and time-of-day variations. While the exact algorithmic components are proprietary, the API produces stable and reproducible travel-time estimates that reflect expected travel conditions. According to the TravelTime documentation, the public transportation data for the study area cover nearly all (95% or more) known stops, and the public transit data coverage is available on the TravelTime Transit Coverage Map (23). This analysis was conducted within ArcGIS Pro (version 3.0.1) using the TravelTime add-in (version 3.0.7).

To estimate hospital service areas, we applied the Time Map Advanced tool with the bus travel model, setting the departure time to 8:00 a.m. on December 16, 2024. We selected this time to represent a typical weekday morning peak period in Austin, when transit demand is high, providing a conservative estimate of hospital service catchment areas and their accessibility during a period when many individuals are likely to rely on public transit for healthcare services. Although results may vary at other times of day or on weekends, we focused on the weekday morning peak for consistency, and future work can extend the analysis to multiple times and days. A 60-min bus travel time was used to define the catchment area, aligning with the widely recognized golden hour standard for healthcare (30, 31). We also set up to 30 min of walking in the transit model to account for access to bus stops and from bus stops to hospitals, reflecting realistic access patterns and consistent with prior evidence that many transit users accumulate 30 min or more of walking per day (32).

### 2.3 Descriptive analysis

We first mapped the spatial distribution of the percentage of the population who are Black or African American, Hispanic or Latino, without health insurance coverage, and with income below the poverty level for each census tract. Next, we overlaid these demographic and socioeconomic data with the hospital service areas accessible within 60 min by public transit to assess geographic disparities in healthcare access among vulnerable populations. To quantify access, we calculated a coverage rate for each census tract, defined as the proportion of the tract’s area that was located within the 60-min hospital service area. This was measured by dividing the area of the tract within the hospital service area by the total tract area. Census tracts were then classified into two categories: no or partial coverage (<100%) and full coverage (100%). These categories were compared with the demographic and socioeconomic characteristics of the tracts to identify how many census tracts with vulnerable populations that face limited access to healthcare via public transit.

## 3 Results

**Figure 1:**
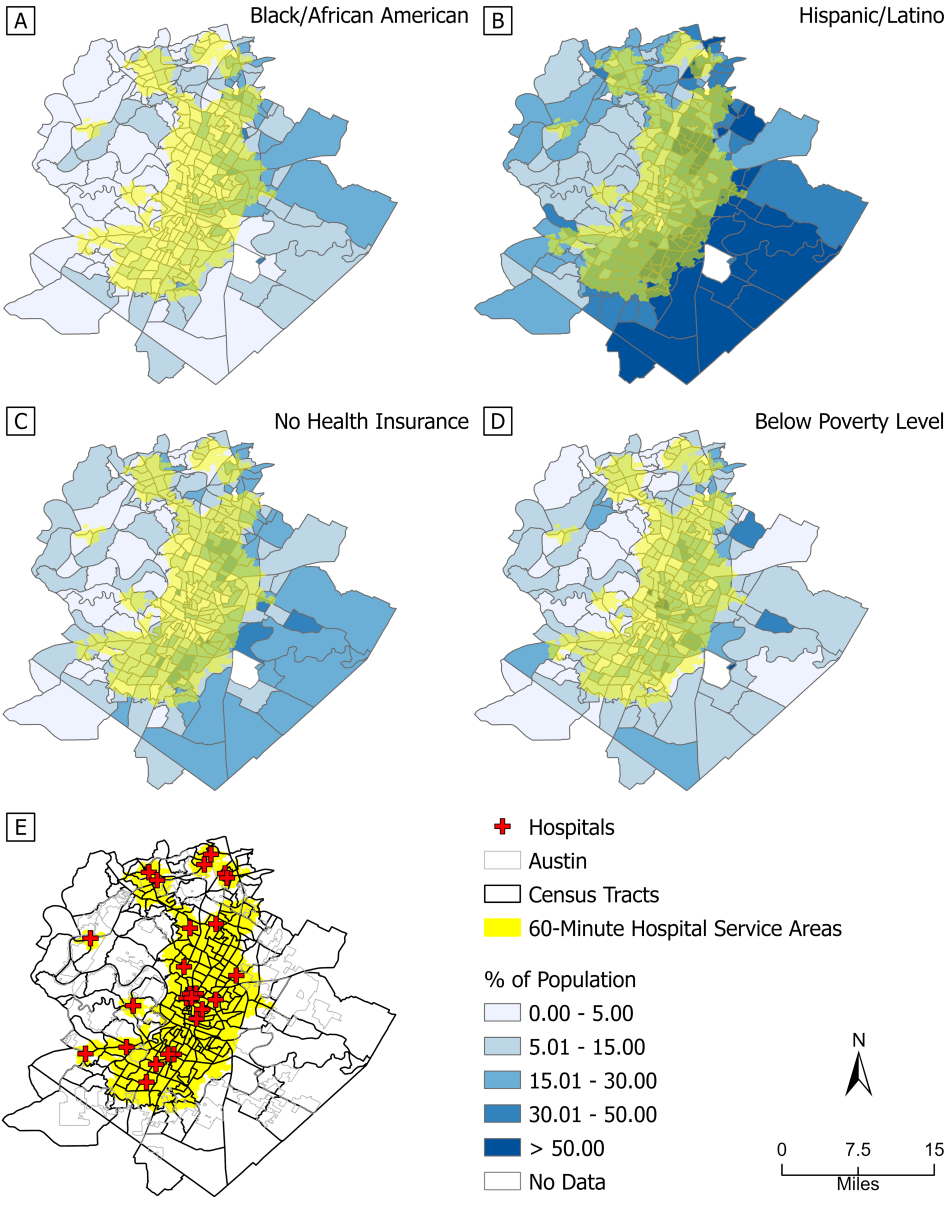
The hospital service area by public transit within 60 min and the percentage of the population. **(A)** Black or African American; **(B)** Hispanic or Latino; **(C)** no health insurance coverage; **(D)** income below the poverty level; **(E)** 60-min hospital service area. Red crosses indicate hospital locations in 2024. Yellow areas indicate hospital service coverage; darker blue colors represent higher population percentages. Sources: Definitive Healthcare; U. S. Census Bureau.

Figure 1 illustrates the spatial distribution of the 60-min hospital service area accessible by public transit in Austin, Texas, alongside demographic and socioeconomic indicators of vulnerability. The maps display the percentage of the population who are Black or African American (Figure 1A), Hispanic or Latino (Figure 1B), uninsured (Figure 1C), and below the poverty line (Figure 1D), with hospital locations marked by red crosses and hospital service catchment areas shaded in yellow (Figure 1E). Darker blue colors represent higher population percentages. The geographical distribution of hospitals (Figure 1E) reveals a clear spatial pattern: hospitals are predominantly clustered in central and north-central Austin, with fewer facilities located in the peripheral regions, particularly in the eastern area.

Of the 283 census tracts analyzed, only 123 were fully covered by the 60-min hospital service area. The remaining 160 tracts were either partially covered (125 tracts) or not covered at all (35 tracts), with 72 of the partially covered tracts having less than 50% area coverage. Overall, compared to central, north-central, and south-central Austin, people living in the western and eastern parts of the city have limited access to hospital services through public transit. Notably, 13 of the 35 uncovered tracts are located in eastern Austin and have Hispanic or Latino populations exceeding 50%. These spatial patterns reveal significant disparities in healthcare access, particularly for vulnerable populations concentrated in eastern Austin. These areas also tend to have higher proportions of the population without health insurance and greater percentages of Black or African American residents. This geographic disparity between hospital accessibility and population need underscores systemic inequities in public transit infrastructure and equitable healthcare access. Detailed demographic characteristics of the 35 uncovered tracts are provided in the Supplementary Table 1, further emphasizing the need for targeted transit and healthcare planning in these underserved areas. While our primary focus was the 60-min threshold to align with the ‘golden hour’ standard, we also generated a supplementary map (Supplementary Figure 1) showing 90-min hospital service catchment areas by public transit to illustrate broader variations in accessibility.

Table 1 compares demographic and socioeconomic disparities between census tracts with no or partial hospital service coverage via public transit within 60 min and those fully covered. Among Black or African American residents, 21 tracts with a total population of 96,768 (7.59%) in the not fully covered area have more than 15% Black or African American population, compared with 21 tracts and 90,588 population (7.11%) in the fully covered area. The disparities are more pronounced for Hispanic or Latino residents: in the not fully covered area, 31 tracts with a total population of 154,838 (12.15%) have more than 50% Hispanic or Latino population, whereas in the fully covered area, 24 tracts with a total population of 106,081 (8.32%) have more than 50% Hispanic or Latino population. Health insurance coverage shows a similar contrast. In the not fully covered area, 40 tracts with a total population of 190,535 (14.95%) have more than 15% of residents uninsured, compared with 41 tracts and a total population of 170,111 (13.34%) in the fully covered area. Poverty levels show a different pattern: in the not fully covered area, 4 tracts with a total population of 20,910 (1.64%) have more than 30% of the population with income below the poverty line, whereas in the fully covered area, 14 tracts with a total population of 57,077 (4.48%) fall into this category. These comparisons indicate that not fully covered areas tend to have higher proportions of racial/ethnic minorities and uninsured residents, where limited transit access to hospitals intersects with socioeconomic disadvantage. These differences were statistically evaluated using two-proportion z tests, which confirmed significant disparities across coverage groups for populations exceeding key demographic thresholds (Supplementary Figures 2–5).

**Table 1:** Summary of census tracts with partial or no coverage and full coverage by the 60-min hospital service area via public transit, categorized by key demographic and socioeconomic characteristics.

Demographic/socioeconomic characteristic	No or partial 60-min hospital service area coverage (160 census tracts)			Within the 60-min hospital service area coverage (123 census tracts)		
	No. of census tracts	Population in census tracts	% of total population	No. of census tracts	Population in census tracts	% of total population
Black/African American, %						
0.00–5.00	83	386,120	30.29	60	229,610	18.01
5.01–15.00	56	297,257	23.32	42	174,560	13.69
15.01–30.00	19	91,429	7.17	19	84,188	6.60
30.01–50.00	2	5,339	0.42	2	6,400	0.50
>50.00	0	0	0	0	0	0.00
Hispanic/Latino, %						
0.00–5.00	1	2,412	0.19	2	6,342	0.50
5.01–15.00	40	184,574	14.48	13	52,435	4.11
15.01–30.00	57	294,708	23.12	42	149,623	11.74
30.01–50.00	31	143,613	11.26	42	180,277	14.14
>50.00	31	154,838	12.15	24	106,081	8.32
No health insurance coverage, %						
0.00–5.00	41	197,912	15.52	23	86,369	6.77
5.01–15.00	79	391,698	30.72	59	238,278	18.69
15.01–30.00	36	173,075	13.58	29	116,148	9.11
30.01–50.00	4	17,460	1.37	11	52,302	4.10
>50.00	0	0	0	1	1,661	0.13
Income below poverty level, %						
0.00–5.00	53	264,542	20.75	16	64,175	5.03
5.01–15.00	84	419,349	32.89	49	199,542	15.65
15.01–30.00	19	75,344	5.91	44	173,964	13.65
30.01–50.00	3	18,652	1.46	8	32,619	2.56
>50.00	1	2,258	0.18	6	24,458	1.92

Population in census tracts: the total number of residents living in census tracts that fall within each demographic/socioeconomic range.

% of total population: the proportion of this subgroup relative to the total population across all 283 census tracts.

## 4 Discussion

This study analyzed disparities in public transit access to hospital services within a 60-min travel time in Austin, Texas, and identified communities with vulnerable populations that face limited access. The results revealed clear spatial inequalities in western and eastern Austin, where no hospital services are reachable within an hour by public transit. The eastern part of the city also has a higher concentration of minority populations and individuals without health insurance. Census tracts with partial or no hospital service coverage consistently show higher proportions of racial/ethnic minorities and uninsured residents. The disparity is especially pronounced among Hispanic and Latino populations, as 31 census tracts with partial or no hospital coverage affect approximately 154,838 people, with more than 50% of them identifying as Hispanic or Latino. To reduce these disparities, city planners should consider expanding public transit infrastructure and improving service frequency in underserved areas. In addition, targeted investments in community-based healthcare services such as mobile health clinics or telehealth services could help bridge the healthcare access gap in the short term.

Previous research has examined emergency medical transport times in Austin, Texas, focusing on ambulance services and self-transport by suspected stroke patients (33). Although Black and Hispanic populations were more concentrated in eastern Austin, the study found no significant differences in average transport times across racial groups. In contrast, our study evaluates access to all types of hospitals via public transit and reveals that a higher percentage of Black and Hispanic residents in eastern Austin are unable to reach hospital services within an hour by bus. A recent report by Smith et al. (34) found that adults without access to a private vehicle, particularly those with low incomes or public health insurance, are significantly more likely to forgo needed healthcare due to barriers in public transit. These findings reinforce the importance of public transit in healthcare accessibility and highlight that disparities in transit-based hospital access reflect broader transportation inequities not captured by studies focused only on emergency or private vehicle transport.

Another study investigated the distribution of transit supply, ride-hailing usage, and vehicle ownership in Austin (35). Their findings showed that downtown Austin has the most accessible public transit resources, which aligns with our results indicating that hospital service areas reachable by public transit are primarily concentrated in the downtown area. Such a spatial pattern likely reflects underlying factors such as population density, urbanicity, and transportation infrastructure. Central areas, which are more urbanized and densely populated, tend to have better transit connectivity and higher demand for healthcare services, making them more favorable locations for hospital placement. In contrast, the eastern part of the city, where vulnerable populations such as Black, Hispanic, and uninsured residents are more concentrated (Figures 1A–C), exhibit limited hospital access via public transit. Our study builds on this by showing that residents in both the western and eastern parts of the city face significant challenges in accessing hospital services through public transit.

Liu et al. (4) analyzed disparities in healthcare accessibility via bus and rail transit in the Chicago metropolitan area and found that Black and Hispanic neighborhoods had significantly worse transit-based access to healthcare compared to White neighborhoods. This supports our findings in Austin, where the eastern part of the city, which has a higher concentration of Black or African American and Hispanic or Latino populations, also lacks adequate hospital access by public transit. Compared to this research, our study contributes a new perspective by incorporating health insurance coverage into the analysis. We found that residents in eastern Austin not only face limited transit access to hospitals but also have a higher percentage of uninsured individuals. Hispanic or Latino individuals make up 32.5 percent of Austin’s population, and 13 percent of the city’s residents lack health insurance (24). This overlap between high concentrations of uninsured individuals and Hispanic or Latino populations in eastern Austin highlights the compounded vulnerability of these communities and emphasizes the need for targeted policy interventions to improve both healthcare access and insurance coverage.

One of the key strengths of our study is that it addresses a gap in public transit research in Austin, Texas, by examining transit access from a healthcare-focused perspective. Specifically, we analyzed how public transportation facilitates access to healthcare services, offering a new angle on both transit and healthcare equity. Additionally, we implemented a new methodological approach by utilizing the Isochrone API to assess public transit travel times. This enabled us to conduct catchment area analysis based on travel time rather than geographic distance, providing a more realistic measure of accessibility. A limitation of this study is its narrow geographic focus, as it only examines the city of Austin. This may limit the generalizability of the findings. Future research is needed to determine whether similar patterns exist in other regions. Additionally, this study evaluates accessibility solely based on public transit; future research may incorporate private and multimodal transportation to assess general healthcare access. Nonetheless, the overall framework developed in this study can be applied to other geographic contexts to further explore disparities in public transit access to healthcare.

## Data Availability

The data analyzed in this study is subject to the following licenses/restrictions: the demographic and socioeconomic data analyzed for this study are publicly available from the United States Census Bureau at https://data.census.gov/. The hospital data analyzed for this study are available by subscription from Definitive Healthcare, a subscription-based healthcare data vendor. Data cannot be shared publicly due to Definitive Healthcare subscription restrictions. Researchers interested in the hospital data supporting the findings of this study can access it by subscribing directly through Definitive Healthcare at https://www.definitivehc.com/. Requests to access these datasets should be directed to United States Census Bureau: https://data.census.gov/. Definitive Healthcare: https://www.definitivehc.com/.
