# Supplementary material for: Disparities in public transit access to healthcare in Austin, Texas
Source: Front Public Health. 2025 Nov 3;13:1689733. doi: 10.3389/fpubh.2025.1689733 (PMC12620499; doi:10.3389/fpubh.2025.1689733)
Supplement: Supplementary file 1 [file Supplementary_file_1.docx]

***Supplementary Material***

**Supplementary Table 1.** Characteristics of 35 census tracts with no 60-minute hospital service coverage through public transit.

| **Census Tracts** | **Total Population** | **Black/African American, %** | **Hispanic/ Latino, %** | **No health insurance coverage, %** | **Income below poverty level, %** |
| --- | --- | --- | --- | --- | --- |
| 48209010815 | 2,138 | 0.00 | 8.14 | 3.65 | 0.90 |
| 48209010912 | 8,887 | 3.70 | 17.84 | 4.95 | 3.60 |
| 48209010923 | 10,288 | 9.14 | 50.03 | 12.36 | 5.90 |
| 48453001921 | 3,737 | 0.43 | 5.19 | 3.85 | 3.20 |
| 48453002215 | 4,197 | 7.05 | 80.65 | 38.69 | 30.70 |
| 48453002216 | 10,008 | 7.46 | 56.90 | 17.11 | 8.60 |
| 48453002221 | 2,677 | 13.90 | 58.20 | 10.68 | 7.40 |
| 48453002319 | 2,258 | 31.93 | 35.16 | 16.87 | 57.80 |
| 48453002434 | 1,935 | 4.75 | 56.90 | 18.35 | 17.80 |
| 48453002436 | 2,551 | 1.18 | 76.52 | 21.56 | 6.40 |
| 48453002446 | 1,287 | 0.16 | 34.50 | 4.84 | 5.80 |
| 48453002449 | 6,928 | 5.96 | 82.07 | 12.46 | 11.50 |
| 48453002450 | 3,845 | 5.07 | 66.42 | 22.89 | 13.50 |
| 48453002452 | 2,787 | 3.77 | 58.88 | 27.41 | 6.90 |
| 48453002453 | 5,518 | 5.73 | 73.36 | 19.63 | 4.60 |
| 48453031400 | 4,577 | 0.44 | 15.53 | 12.82 | 10.80 |
| 48453031500 | 7,661 | 3.07 | 20.74 | 14.97 | 6.10 |
| 48453032900 | 8,218 | 0.52 | 14.13 | 1.41 | 3.60 |
| 48453033300 | 9,426 | 6.05 | 12.34 | 5.07 | 5.70 |
| 48453033900 | 5,654 | 1.24 | 13.35 | 3.15 | 0.70 |
| 48453034000 | 6,765 | 1.18 | 9.34 | 6.59 | 7.20 |
| 48453034100 | 6,342 | 12.55 | 20.10 | 15.58 | 9.20 |
| 48453034400 | 2,199 | 4.96 | 20.65 | 8.87 | 15.20 |
| 48453034500 | 2,412 | 0.00 | 1.53 | 5.18 | 23.70 |
| 48453034600 | 4,812 | 10.10 | 27.08 | 2.97 | 15.70 |
| 48453035100 | 4,102 | 7.70 | 8.26 | 0.74 | 4.60 |
| 48453035600 | 2,563 | 0.66 | 11.47 | 5.42 | 3.30 |
| 48453035700 | 4,434 | 0.00 | 14.93 | 7.10 | 6.50 |
| 48453036700 | 7,564 | 0.97 | 28.69 | 1.07 | 1.60 |
| 48453036800 | 4,278 | 4.79 | 9.09 | 1.31 | 3.00 |
| 48453043800 | 3,961 | 14.72 | 56.85 | 22.14 | 7.90 |
| 48453044700 | 1,741 | 6.03 | 62.72 | 22.40 | 0.00 |
| 48453044900 | 10,904 | 8.87 | 56.80 | 23.39 | 30.60 |
| 48453045000 | 2,379 | 18.33 | 33.46 | 13.45 | 6.50 |
| 48491020411 | 5,329 | 9.63 | 16.96 | 8.58 | 4.80 |


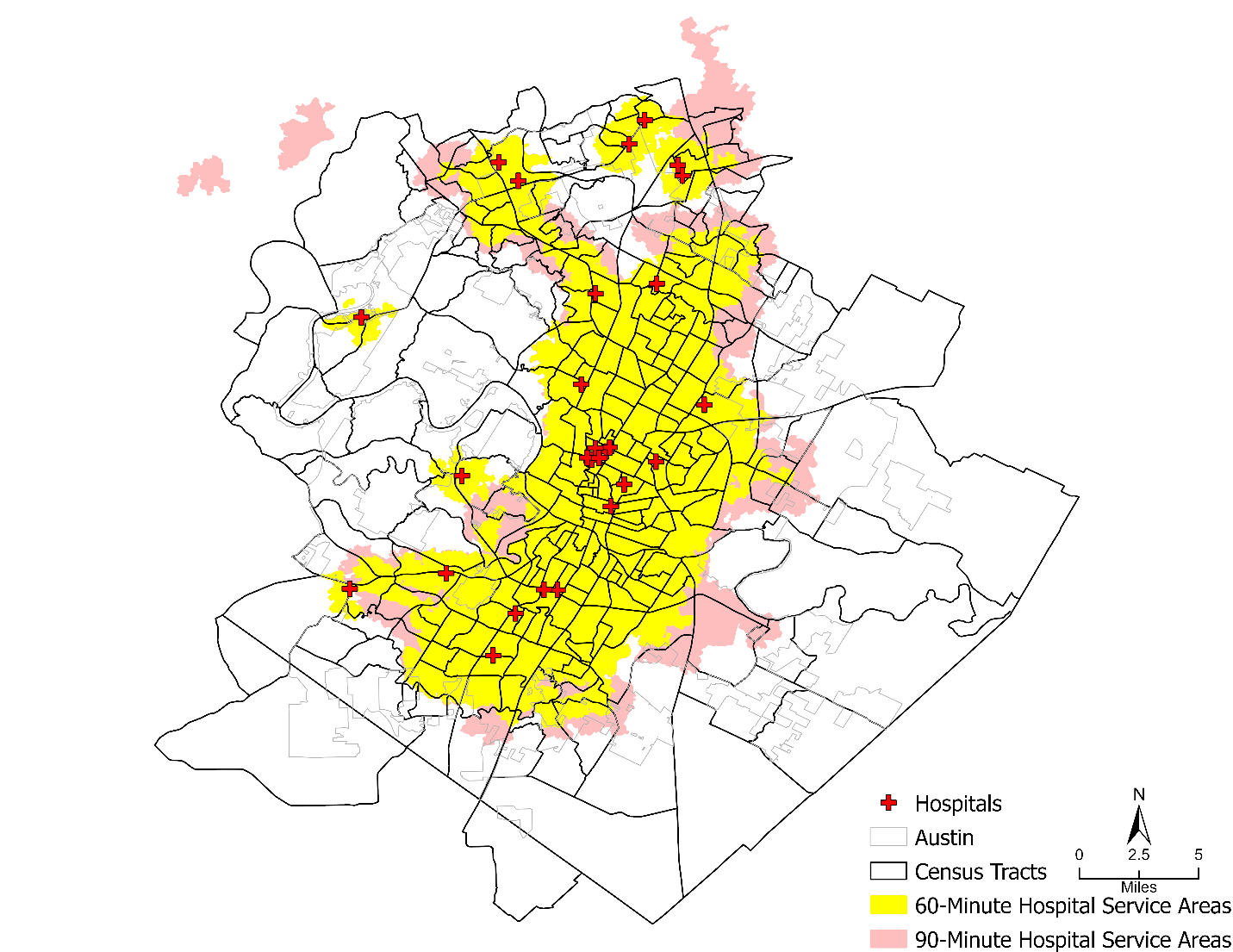


**Supplementary Figure 1.** Hospital service catchment areas by public transit at 60- and 90-minute thresholds.


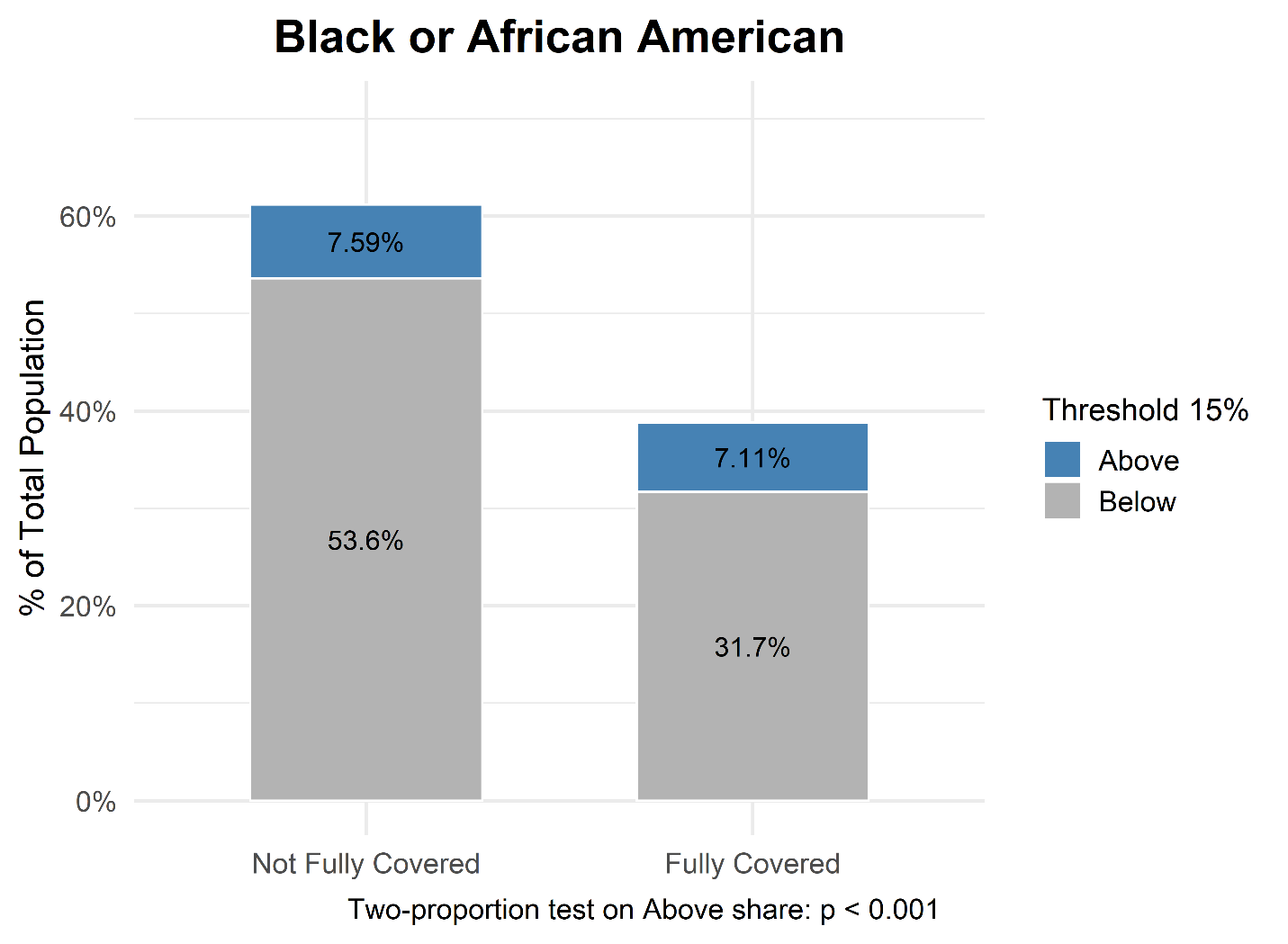


**Supplementary Figure 2.** Distribution of the total population across census tracts with no or partial and full coverage by the 60-minute hospital service area via public transit, categorized by Black or African American group (threshold 15%). Bars represent the percentage of the total population falling above and below the threshold. A two-proportion test (p < 0.001) indicates that the difference between coverage groups in the population above the threshold is statistically significant.


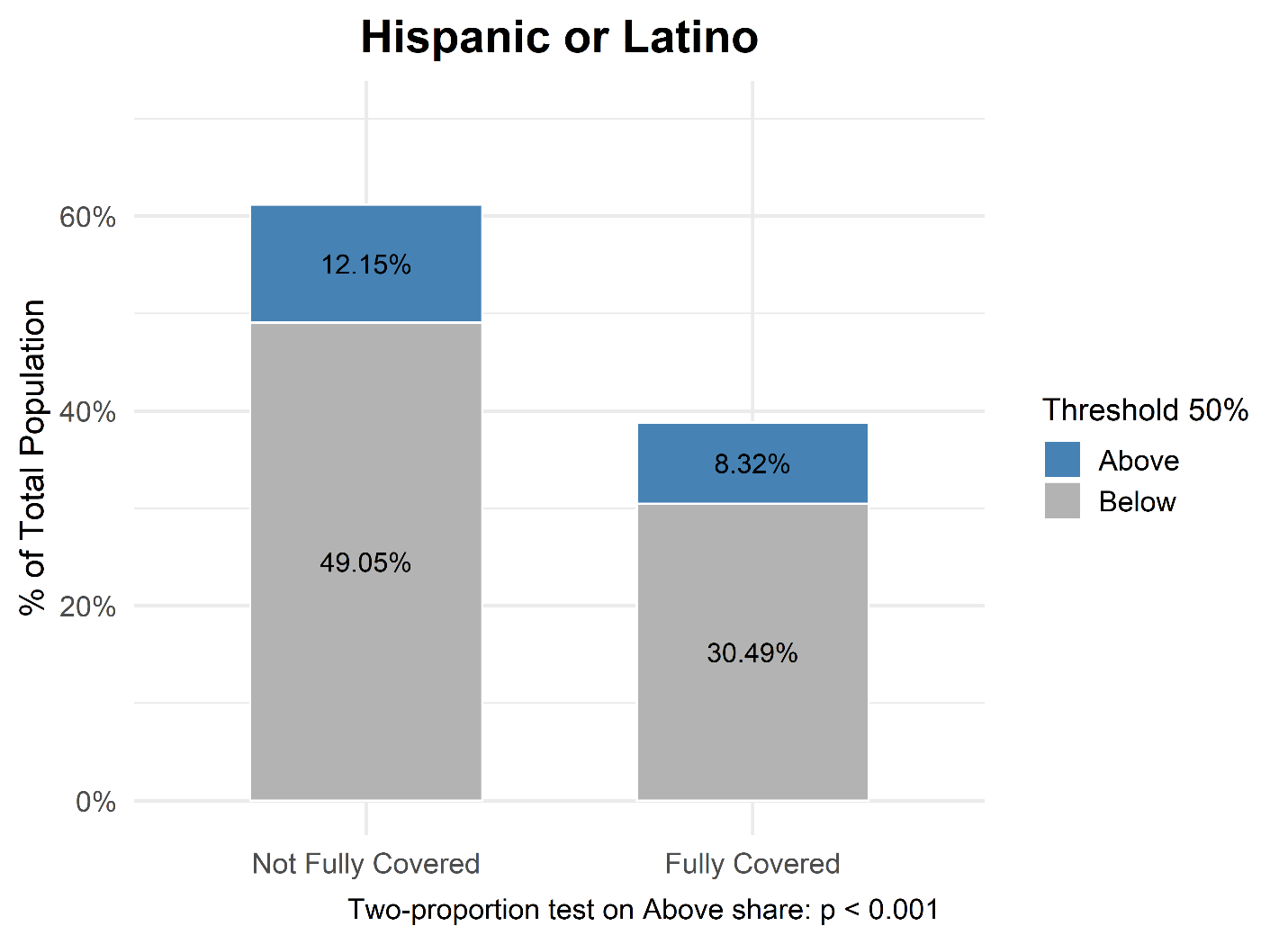


**Supplementary Figure 3.** Distribution of the total population across census tracts with no or partial and full coverage by the 60-minute hospital service area via public transit, categorized by Hispanic or Latino group (threshold 50%). Bars represent the percentage of the total population falling above and below the threshold. A two-proportion test (p < 0.001) indicates that the difference between coverage groups in the population above the threshold is statistically significant.


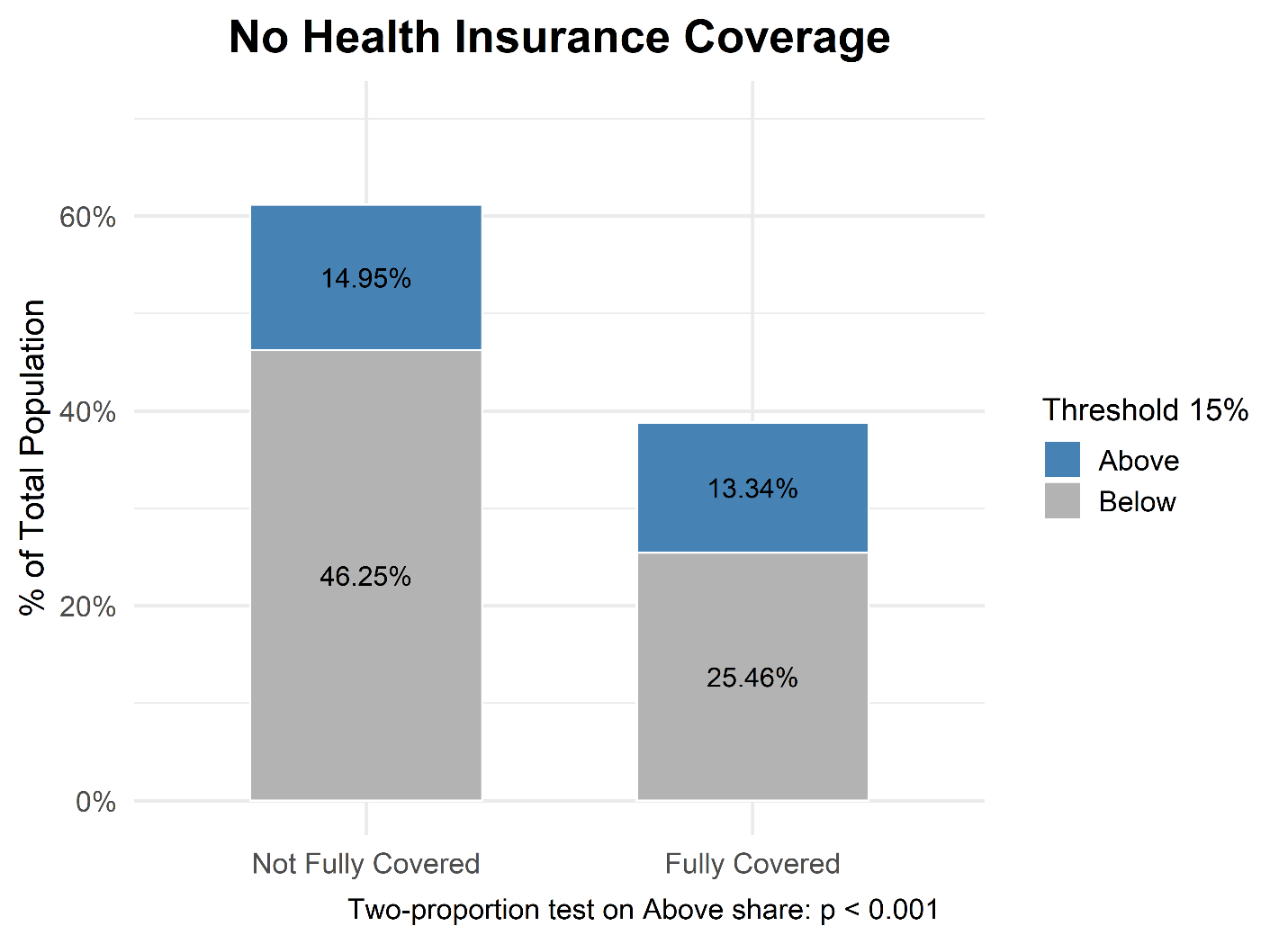


**Supplementary Figure 4.** Distribution of the total population across census tracts with no or partial and full coverage by the 60-minute hospital service area via public transit, categorized by no health insurance coverage (threshold 15%). Bars represent the percentage of the total population falling above and below the threshold. A two-proportion test (p < 0.001) indicates that the difference between coverage groups in the population above the threshold is statistically significant.


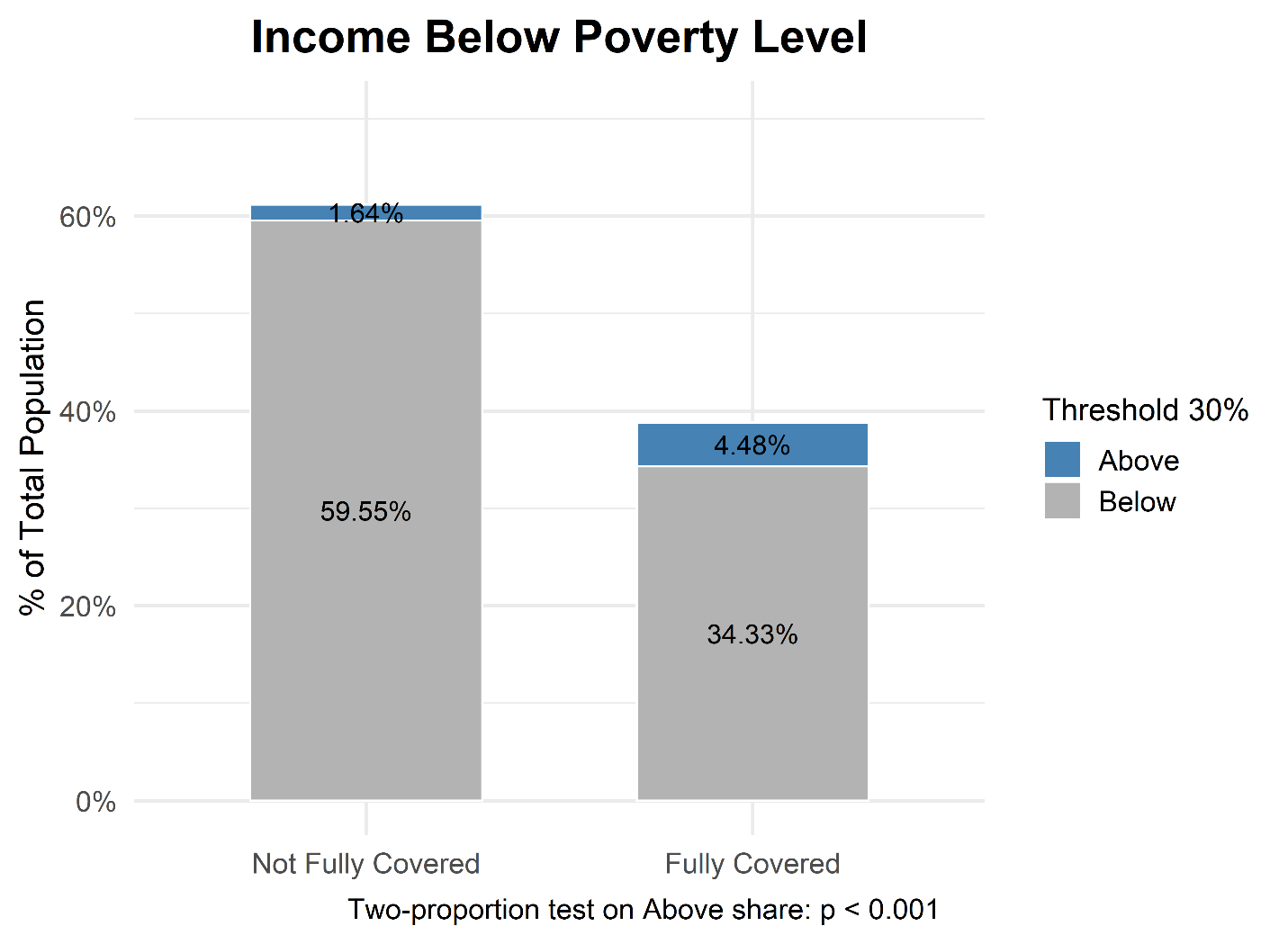


**Supplementary Figure 5.** Distribution of the total population across census tracts with no or partial and full coverage by the 60-minute hospital service area via public transit, categorized by income below the poverty level (threshold 30%). Bars represent the percentage of the total population falling above and below the threshold. A two-proportion test (p < 0.001) indicates that the difference between coverage groups in the population above the threshold is statistically significant.
